# The meaning of dignity in care during the COVID-19 pandemic: a qualitative study in acute and intensive care

**DOI:** 10.1186/s12904-023-01311-4

**Published:** 2023-11-30

**Authors:** Loredana Buonaccorso, Ludovica De Panfilis, Harvey Max Chochinov, Gianfranco Martucci, Marco Massari, Monica Cocchi, Maria Chiara Bassi, Silvia Tanzi

**Affiliations:** 1 Psycho-Oncology Unit, Azienda USL-IRCCS di Reggio Emilia, Reggio Emilia, Italy; 2 Legal Medicine and Bioethics, Azienda USL-IRCSS di Reggio Emilia, Reggio Emilia, Italy; 3 Department of Psychiatry, University of Manitoba, Winnipeg, Canada; 4 Paul Albrechtsen Research, Cancer Care Manitoba, University of Manitoba, Winnipeg, Canada; 5 Local Network of Palliative Care, AUSL Modena, Modena, Italy; 6 Infectious Diseases Unit, Azienda USL-IRCSS di Reggio Emilia, Reggio Emilia, Italy; 7 Hospital Infections Office, Hospital Medical Directorate, Azienda USL-IRCSS di Reggio Emilia, Reggio Emilia, Italy; 8 Medical Library, Azienda USL-IRCSS di Reggio Emilia, Reggio Emilia, Italy; 9 Palliative Care Unit, Azienda USL-IRCSS di Reggio Emilia, Reggio Emilia, Italy

**Keywords:** Dignity, Meaning, COVID-19, Intensive care, Palliative care

## Abstract

**Background:**

The pandemic Era has forced palliative care professionals to use a dignity-in-care approach in different settings from the *classic* ones of palliative care: acute and intensive care. We explored the meanings of dignity for patients, their family members, and clinicians who have experienced COVID-19 in the acute and intensive care setting.

**Methods:**

A qualitative, prospective study by means of semi-structured interviews with patients hospitalized for COVID-19, family members, and clinicians who care for them.

**Findings:**

Between March 2021 and October 2021, we interviewed 16 participants: five physicians, three nurses, and eight patients. None of the patients interviewed consented for family members to participate: they considered it important to protect them from bringing the painful memory back to the period of their hospitalization. Several concepts and themes arose from the interviews: humanity, reciprocity, connectedness, and relationship, as confirmed by the literature. Interestingly, both healthcare professionals and patients expressed the value of *informing* and *being informed* about clinical conditions and uncertainties to protect dignity.

**Conclusions:**

Dignity should be enhanced by all healthcare professionals, not only those in palliative care or end-of-life but also in emergency departments.

**Supplementary Information:**

The online version contains supplementary material available at 10.1186/s12904-023-01311-4.

## Background

During the COVID-19 outbreak, hospital care systems had to reorganize their activities. The changes imposed by contagion containment rules have radically altered how patients are cared for [1–3]. Several articles have been published in recent years on the ethics of dignity and the spiritual and psychological suffering of patients hospitalized for COVID-19 and their families [4–8].

“Respecting dignity” emerged as a moral and ethical imperative, as the respect for the intrinsic value of human beings and the patient’s autonomy and choices [6]. A qualitative study with bereaved relatives of patients who died during the COVID-19 pandemic between March and July 2020 in the Netherlands showed that the issue of dignity during end-of-life care had to be understood in new ways and new contexts [7].

A dignity-in-care-based approach also emerged as meaningful in the emergency setting before the COVID-19 outbreak, showing that small measures, such as special sensitivity and attention to emotional matters, enhance the patient’s dignity together with the efforts to manage all symptoms [9–11].

The studies described above were conducted from the healthcare professional (HPs) perspective, but no patients’ experiences were investigated. Some of these studies [7–11] were inspired by the Model of Dignity for terminally ill patients, derived from the studies of Chochinov and colleagues [12].

Our Specialized and multi-professional Palliative Care Service has been involved in dignity therapy studies [13–15] and interventions over the last three years. A study was conducted on dignity therapy in different care settings (home care and the oncology hospital) and during different stages of cancer [13]. Also, a dignity therapy training and implementation experience, exploring the potential enablers/barriers to implementing these types of interventions, was embarked [15]. The service also experimented with dignity therapy with culturally and linguistically diverse cancer patients, particularly those from Muslim backgrounds [14]. In response to the pandemic and building on the previous experiences with oncological patients, ideal practices for preserving dignity have been reviewed and then implemented, as well as for self-awareness, compassionate presence, and spiritual care [16]. Moreover, the pandemic Era forced the use of the dignity-in-care approach in different settings from the *classic* ones of palliative care (PC): acute and intensive care. Several departments that assisted patients with COVID-19 have been supported in teaching colleagues the principles of PC. In particular, the importance of how to preserve dignity even when there are clinical uncertainties or when time is limited to a few minutes has been “touched” [16–19]. This included a commitment “*to stay*” i.e., to not abandon patients, to treat symptoms properly, to avoid inappropriate or harmful interventions, and to provide relief from suffering are ways to safeguard dignity [19, 20].

To our knowledge, no study has been conducted to investigate the construct of dignity in patients who have experienced COVID-19, their families, and the HPs who have assisted them in acute and intensive care settings.

### Objectives

The study explores the meanings of dignity for patients, their family members, and HPs who have experienced COVID-19 in the acute and intensive care setting.

## Methods

### Design

A qualitative, prospective study by means of semi-structured interviews with:

i) patients hospitalized for COVID-19,
ii) family members of patients who have been hospitalized for COVID-19,
iii) HPs (physicians and nurses) caring for hospitalized patients with COVID-19.

The consolidated criteria for reporting qualitative research (COREQ) guided the reporting of our findings (Supplementary File 1).

The Braun and Clarke’s methodological framework [21] has been followed, considering the theoretical approach suggested originally in Guba and Lincoln (1989) and revisited by Luciani and Campbell (“How to critically appraise a qualitative health study”) [22]. The constructivist approach has been chosen as the epistemological stance, as the study tries to understand a specific and peculiar experience lived by the subjects, producing ad interpretation of data gathered through multiple perspectives.

### Setting and recruitment

The study was conducted at the “Arcispedale Santa Maria Nuova” General Hospital, a 900-bed public hospital located in Reggio Emilia, in northern Italy, which was recently designated as a Comprehensive Clinical Cancer Center by the Organization of European Cancer Institutes (OECI).

### Sample

A purposive sample of HPs from the Infectious Diseases Department was involved, together with patients who were hospitalized for COVID-19 in this Department between March 2020 and October 2021. The heterogeneity of the patient population (who received ventilation and non-ventilation intervention) and the intensity of intervention in acute and intensive care were considered.

Specifically, according to inclusion criteria, six physicians and seven nurses were randomly selected and contacted by GM using a list of names agreed upon with the head of the Department and the Head of Nursing. A list of ten patients was provided by the Head of Nursing, while family members were suggested by patient participants. The family members are those who have an informal role of care, support and closeness and who participate in the patient’s illness experience and who engage in daily personal care activities.

The inclusion criteria were:

1. HPs who assisted patients during COVID-19 for at least one week,
2. hospitalized patients who experienced COVID-19 and who have not been seen by or require support from the Emergency Psychology Service,
3. family members of living patients hospitalized for COVID-19 and who have not been seen by or require support from the Emergency Psychology Service,
4. all aged > 18.

From March 2020 to October 2021, physicians and nurses cared for 2,423 COVID-19 patients.

Between March 2021 and October 2021, we selected 13 HPs and ten patients, as described previously in the “sample” paragraph.

**Fig. 1:**
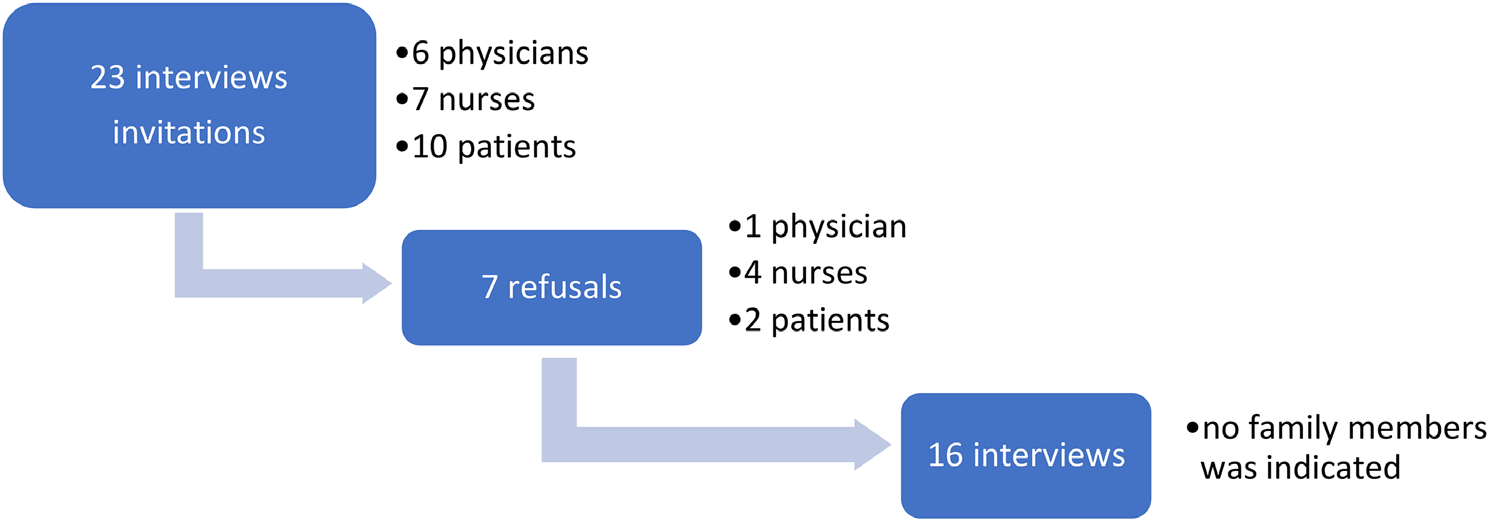
The path for the selection of participants

Figure 1 shows the path of selection for participants.

We interviewed 16 participants: five physicians, three nurses, and eight patients (Tables 1 and 2 provides the participants’ sociodemographic characteristics). None of the patients interviewed consented for family members to participate: they considered it important to protect them from bringing the painful memory back to the period of their hospitalization.

In particular, none of the patients expected to be interviewed on the subject of dignity. All patients told the interviewers that they had appreciated an interview on this topic and that they were able to remember difficult moments in a “protected” context where they felt supported.

Two interviews were conducted via audio call and 14 by video call; all lasted between 15 and 40 min.

### Data collection

The individual interviews were conducted by video or audio call. All of the interviews were audio-recorded and transcribed verbatim.

The interviews were developed for this study (Supplementary File 2). A multi-disciplinary expert panel––consisting of a researcher and psycho-oncologist psychotherapist (LB), a PC researcher and bioethicist (LDP), a researcher and PC physician (ST), and a researcher physician (GM)––designed the interviews, after reviewing the literature on the concept of dignity in terminally ill patients. Some questions of the interviews was based on the study by Chochinov et al. that examined perceptions of dignity in patients nearing death [12]. We decided to use this model as a reference because it took the experience of patients into direct consideration. In addition, the research team is familiar with this model and employs it in clinical practice [13–16]. The interview explored how patients and families perceived dignity, and how HPs perceived the patients’ and their own dignity during the COVID-19 assistance.

The semi-structured interview let the interviewers the chance to rephrase and adapt the question to the single interviewee, specifying terms or clarifying concepts as needed. At the end of each interview, they met with a third researcher (GM) to supervise the progress of the interview.

A member of the research team (GM) contacted each patient to ask for consent to be interviewed. Two authors (LB and LDP) conducted the interviews. Before the start of each interview, written and verbal informed consent was obtained from all participants. Attention was paid to after-care, in particular, in light of the type of questions that would investigate the experience linked to hospitalization for COVID-19, participants were informed that they could speak to a dedicated psychologist if needed following the interview.

### Data analysis

One author (LB) transcribed the interviews, and two authors (LDP and ST) conducted the thematic analysis based on the verbatim transcripts. The data was validated by two external researchers (LB and GM).

Interviews were analyzed as a unified dataset comprised of all interviews. The analysis process consisted of several steps, according to Braun and Clarke [21]. All researchers received a detailed guide on how to approach the analysis according to the thematic analysis. They all went through the different stages required, which were (1) familiarizing oneself with the data, (2) generating initial codes, (3) searching for themes (4) reviewing themes and (5) defining and naming them, and finally (6) the production of an initial report.

This process was designed to lead to the development of a thematic map (which aimed to visually represent the relationship between the codes/themes and the data set), a list of themes with a short definition of each theme, comprehensive inclusion and exclusion criteria (answering the question “Why does this theme include this tag and not that tag?”), and a selection of significant quotations.

The two thematic analyses were then compared to obtain a third, more robust analysis, under the supervision of a third researcher (GM). The need to reach data saturation was discussed, but was deemed not important in light of the type of interview and the research question. We used the work of Guba and Lincoln [22] to focus on *credibility, dependability*, and *confirmability*. To increase the *credibility* of our analysis we used *triangulation of sources* (as we interviewed subjects with different roles, such as HPs and patients, about the same experience), and *triangulation of researchers* (researchers with different backgrounds, professions, and involvement in the study, e.g., two external researchers did not conduct the interviews) [23]. *Researchers’credibility* was also quite relevant, as all of the researchers involved in the process had a PC background, including publications on the topic of dignity-in-care, and were familiar with the ward dynamics and routines. We also focused on the *dependability* of our analysis, based on *achieving consensus among different coders.* Furthermore, we attempted to increase the *confirmability* of our research by way of *reflexivity* [23].

### Reflexivity

The interviewers, moderators, and analysts were experts in qualitative methods and were supervised by a researcher physician (GM). The authors consisted of a researcher and psycho-oncologist psychotherapist with PC experiences (LB), a PC researcher and bioethicist (LDP), a researcher and PC physician (ST), and a researcher physician (GM). The interdisciplinary analysis obviated personal interests or disciplinary assumptions. The interviewers (LB and LDP) did not have any pre-existing relationship with the participants and were external to the work settings of the HPs. They are specifically trained in conducting qualitative interviews. Moreover, they had a PC background, including published papers on the topic of dignity therapy, and they were all familiar with the ward considered. Finally, to increase the confirmability of the results, an author (HMC), external to the data gathering and analysis, provided relevant methodological feedback, and an audit trail of the analysis process.

## Findings

Three major categories emerged from the qualitative analysis, including: (1) Dignity in and of itself, (2) Dignity in relationships, and (3) Dignity in practice. Each of these categories contains various themes and sub-themes (Table 3). Exemplars for each of these themes and sub-themes illustrate the concepts contained therein. Major-theme exemplars and quotations are reported in Table 4.

**Table 1:** The sociodemographic characteristics of the Healthcare Professionals

Healthcare Professionals	Age	Gender	Civil status	Education	Religious belief and involvement	Years of work	Care Department
Physician 1	57	Female	Separated/Divorced	Post-graduated school	Believer, does not attend religious services	28	Infectious Disease
Physician 2	32	Female	In a relationship	Post-graduated school	Attends religious services	6	Infectious Disease
Physician 3	32	Male	Single	Post-graduated school	Believer, does not attend religious services	1	Infectious Disease
Nurse 4	27	Female	Single	Post-graduated school	Attends religious services	4	Infectious Disease
Physician 5	32	Male	Single	Post-graduated school	Non-believer	4	Infectious Disease
Nurse 6	25	Female	Single	Post-graduated school	Believer, does not attend religious services	1	Infectious Disease
Nurse 7	32	Female	In a relationship	Post-graduated school	Believer, does not attend religious services	5	Infectious Disease
Physician 8	32	Male	In a relationship	Post-graduated school	Believer, does not attend religious services	6	Infectious Disease

**Table 2:** The sociodemographic characteristics of the patients

Patients	Age	Gender	Civil status	Education	Religious belief and involvement	Occupation	Care Department	Type of clinical intervention
Patient 1	50	Female	In a relationship	Middle school diploma	Believer, does not attend religious services	Employed	Infectious Disease Emergency department	Oxygen goggles
Patient 2	56	Female	In a relationship	Middle school diploma	Non-believer	Employed	Infectious Disease Emergency department	Non-invasive ventilation
Patient 3	66	Female	In a relationship	Middle school diploma	Believer, does not attend religious services	Retired	Infectious Disease	Non-invasive ventilation
Patient 4	45	Female	In a relationship	High school diploma	Non-believer	Unemployed	Infectious Disease Medicine	Oxygen goggles
Patient 5	50	Male	Single	University degree	Attends religious services	Employed	Infectious Disease Pneumology department Medicine	Invasive ventilation
Patient 6	23	Female	Single	High school diploma	Believer, does not attend religious services	Student	Infectious Disease Medicine	Non-invasive ventilation
Patient 7	53	Female	Separated/Divorced	High school diploma	Attends religious services	Employed	Infectious Disease Pneumology department Intensive care	Invasive ventilation
Patient 8	63	Male	In a relationship	Middle school diploma	Attends religious services	Employed	Infectious Disease	Oxygen goggles

**Table 3:** Major themes: Categories, themes, and sub-themes of the construction of dignity in the acute and intensive care setting

Categories	Dignity in and of itself “Moral and inalienable good of all human beings”	Dignity in relationships “Being present with self-awareness”	Dignity in practice “Tone of care”
**Themes**	**Dignity as a Value**	**Fragility**	**Preservation of Choice**
**Subthemes**	- Values - Beliefs - Culture - Morality	- S*taying* in a relationship - *Being* present with self-awareness - Self-care	**-***Informing* and *being informed*
**Themes**	**Sense of Intactness**	**Reciprocity**	**The Body and Small Everyday Acts**
**Subthemes**	- Human person - Autonomy - Consciousness - Emotions - Freedom	- One’s own attitude and assumptions influence dignity - Dignified actions	- Small things - Acts - Time
**Themes**	**Humanity**	**Deep Understanding**	**Information Overload**
**Subthemes**	- Caring for the person (not only the disease)	- Empathy - Compassion	- Daily information for patients and families
**Themes**		**Connectedness**	
**Subthemes**		- “Hospital system” and “patient system” close together	

### Dignity in and of itself

Dignity emerges as a category unto itself. It refers to the “moral and inalienable value of all human beings”. The importance of respecting patients’ values, beliefs, culture, and morality highlights the fact that dignity is inseparable from the person themself. Three themes within this category are “Dignity as a Value,” “Sense of Intactness,” and “Humanity”.

#### Dignity as a value

Dignity emerges with a dual meaning: the values and preferences of the other, which are to be respected, and one’s own values and principles, to be affirmed and preserved in all circumstances. In our analyses, the participants reported that dignity is innate in the human being and an inalienable right. At the same time, recognizing those aspects confers dignity by respecting and honoring that individual.

#### Sense of intactness

The meaning of dignity in care includes the appreciation of the human person as an intact whole:

“*I believe that if someone lost their dignity, to me they would no longer make sense as a person (…). To live without dignity strips away personhood”* (Nurse 4).

This sub-theme seems to be dependent on how others view or perceive people in the care relationship. Loss of dignity implies the loss of the human person as an intact being, meaning a loss of health, autonomy, consciousness (being present to oneself), emotions, and freedom (in the case of long hospitalizations). The restoration of dignity is enabled when HPs return this wholeness to patients by making eye contact with them, giving them back their autonomy, giving them the information that allows them to choose (supporting their rejecting of certain treatments), allowing them to express their emotions, etc.

#### Humanity

Humanity is the state of being humane. The dignity of the patient is respected while maintaining the human part of care, not only the medical dimension:

*“Dignity is doing what is necessary to care for people, giving care to the patients we have, and taking into consideration the humanity, the part of the patient that is personal”* (Physician 2).

Dignity enriches the concept of humanity. Dignity means seeing the patient as a human person, not only as an object of care. This includes dedicating time to speak with patients in ways that affirm who they are as a person. Respecting dignity means being recognized as a person.

### Dignity in relationships

The second category describes the link between dignity and how the care relationship is experienced. The essence of this relationship is “being present with self-awareness”. The themes include, “Fragility,” “Reciprocity,” “Deep Understanding,” and “Connectedness”.

#### Fragility

The extreme fragility of patients with COVID-19 and HPs traced back to a lack of knowledge about therapies and treatments, the lack of experience of HPs supporting terminally ill patients through the PC approach, the large numbers of patients, and the patients’ isolation. Moreover, while patients live in fear of a virus that can be fatal, HPs struggle with the risk of exposure and contamination. The fragility perceived in patients and felt by HPs brings out the importance of the care relationship. Attending to fragility means first of all recognizing it in patients and oneself as HPs, so that every gesture of care, from therapies to emotional support, can be optimally expressed.

#### Reciprocity

It came to light that dignity is something that HPs and patients can give or take from each other. The participants reported that dignity is a *personal concept* that is shaped by life experiences (educational, social, and cultural factors), but it can be influenced by how others react to/interact with you:

“*You may sometimes conduct yourself in a dignified manner, then you find yourself in a given situation that, conversely, deprives you of your dignity… In such a circumstance, it is normal that your dignity would have to be reset. That’s what I think”* (Patient 6).

#### Deep understanding

Conveying a deep understanding to the patient of their experience, taking part in their healing, and sharing in the care relationship, allows the patient to feel their own dignity:

“*Understanding what the patient is going through (…) taking into account what a person––one with a serious illness––is feeling (…)“dignity” includes an understanding of the feelings of the individual”* (Patient 3).

#### Connectedness

Participants say that being hospitalized and dying without family denied the maintenance of dignity. The lack of relationships and remaining isolated in a room while waiting to worsen or recover undermined personal dignity. Conversely, the absence of family members has increased empathy and sharing between patients and HPs:

“*All of the HPs were very attentive from a human standpoint. They were supportive of the patients, in an extremely empathetic and intense way, and that is a wonderful thing”* (Physician 3).

### Dignity in practice

The third category translates the essences of the other two categories, “Dignity in and of itself” and “Dignity in relationships,” into clinical practice. It could be described as the tone of care [12]. The patients reported that their dignity could be safeguarded or undermined at any time despite their fragile condition (the result of clinical uncertainty and isolation). The themes include, “Preservation of Choice,” “The Body and Small Everyday Acts,” and “Information Overload”.

#### Preservation of choice

In the interviews, both HPs and patients expressed the value of *informing* and *being informed* about clinical conditions, uncertainties, and communication difficulties related to COVID-19. Patients felt their dignity was violated when they were unable to choose therapies e.g., the type of ventilation. In particular, some patients equated the lack of information to a lack of respect for their dignity. This was mirrored by the feelings of HPs who, when they were able to correctly inform patients, perceived that they had respected their dignity.

#### The body and small everyday acts

According to HPs and patients, dignity can be conveyed through small acts, attention, and eye contact. On the other hand, denying the patient these things (e.g., not attending to their hygiene for a long time) is perceived as highly detrimental to dignity.

For HPs, COVID-19 has demonstrated the importance of learning, small acts, and the giving of time.

#### Information overload

Both HPs and patients felt the amount of information received about COVID-19 had a significant role in the relationship between daily care and the perception of each other’s dignity. They felt that information overload could undermine a sense of hope and make a patient feel besieged by negative thoughts. Consequently, HPs also felt that their role was undermined by a partial information overload, e.g., regarding treatments or hospital disorganization.

**Fig. 2:**
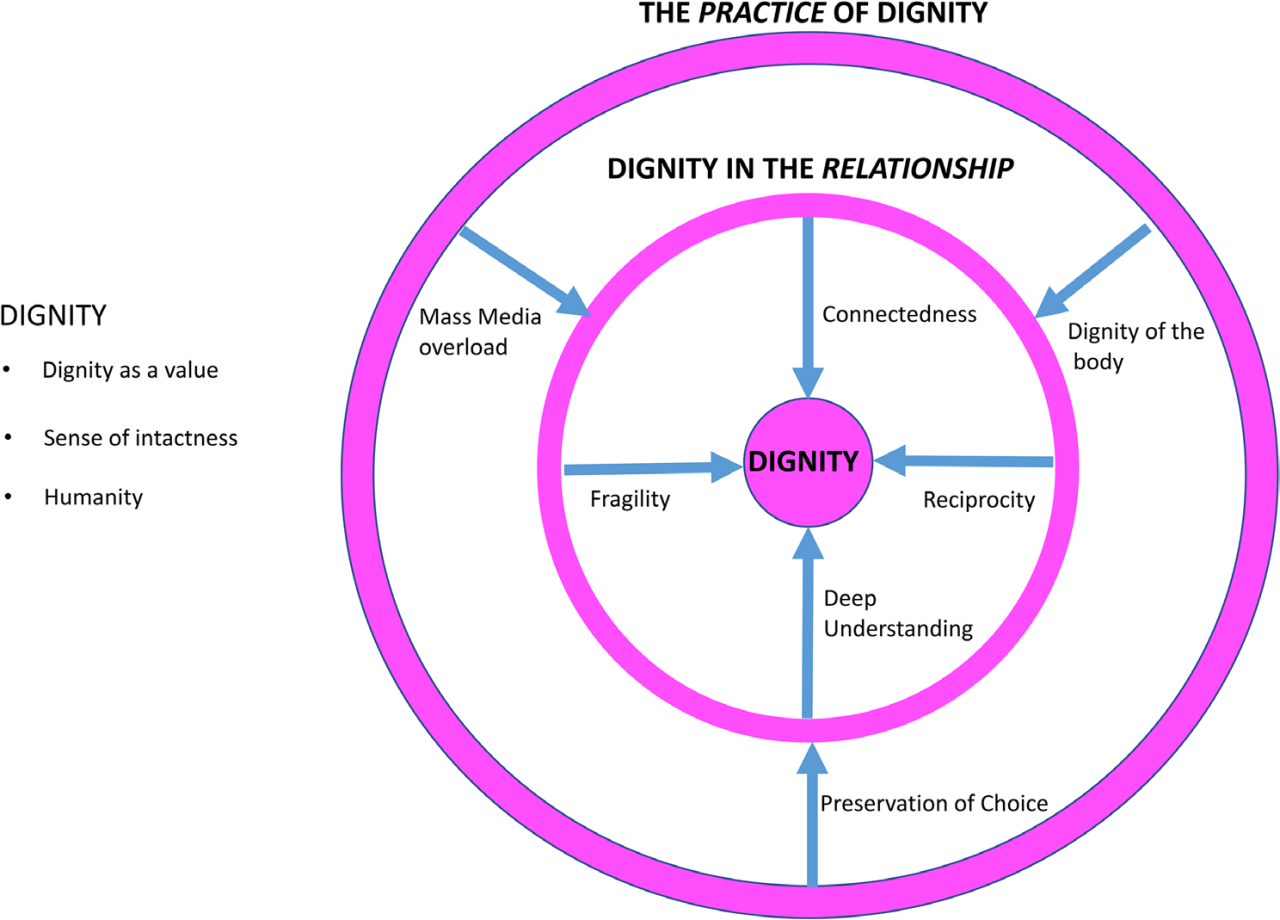
Major themes and tools to support communication between patients and healthcare professionals to enhance reciprocal dignity in the acute and intensive care setting

Figure 2 describes major themes and tools to support communication between patients and HPs to enhance reciprocal dignity in acute and intensive care settings. The arrows are all oriented towards “core” dignity because they are the elements/practices that restore dignity to the patient (according to our results).

**Table 4:** Major Theme Exemplars and Quotations

Category 1	Themes	Exemplars and Quotations
**Dignity in and of itself**	**1.1 Dignity as a Value**	*“Respect for the person, their beliefs (…) therefore dignity is doing the least possible that would disturb a person, their beliefs, their values, their culture.“* (Patient 4) *“It is equally important not to lose dignity.“* (Nurse 4) *“Each of us has our dignity to maintain whenever and wherever we are.“* (Nurse 6)
	**1.2 Sense of Intactness**	*“I believe that if someone lost their dignity, to me they would no longer make sense as a person. (…) To live without dignity strips away personhood.”* (Nurse 4) *“To have dignity, it is important to be treated as a person, not as a COVID-19-positive patient.“* (Patient 1)
	**1.3 Humanity**	“*Knowing that you have been important from a human point of view.”* (Patient 2) *“More than dignity, it was actual humanity, especially if we consider everything that they needed to pay attention to.”* (Patient 6) *“I believe that dignity is the meaning of life; it is what a person can have, which is, to be dignified”* (Patient 6) *“Dignity is doing what is necessary to care for people, giving care to the patients we have, and taking into consideration the humanity, the part of the patient that is personal, and not only the disease on a scientific basis. For there to be dignity, there must also be a human and personal part, in my opinion.”* (Physician 2)”
**Category 2**	**Themes**	**Exemplars and Quotations**
**Dignity in relationships**	**2.1 Fragility **	*“(…) In those moments you are bound to them, you depend on them, you are in their hands. In the end, if I am still alive, I have them to thank for it.“* (Patient 6) *“Difficulties related to exhaustion, to tiredness, to the fatigue from seeing so many patients, to no longer being able to listen because you can no longer physically cope with it, combine with the additional difficulty of not having the tools or adequate knowledge.“* (Physician 5)
	**2.2 Reciprocity**	*“You may sometimes conduct yourself in a dignified manner, then you find yourself in a given situation that, conversely, deprives you of your dignity… In such a circumstance, it is normal that your dignity would have to be reset. That’s what I think.“* (Patient 6) “*Dignity came from the patients, from their thanks, from the acknowledgment that, in any event, what we had done had helped them, including from an emotional point of view.“* (Physician 5)
	**2.3 Deep Understanding**	*“Understanding what the patient is going through (…) taking into account what a person––one with a serious illness––is feeling (…) “dignity” includes an understanding of the feelings of the individual.“* (Patient 3) *“When they had the final ‘okay’ (for my discharge), a nurse came in (too bad I don’t remember her name). She came in and said to me ‘Anto, you’re going home! Anto, you’re going home! I’ll call you an ambulance!’. What happiness. What happiness! It felt like a party, because everyone––each and every one of the people working––were all cheerful, because they had found out that the doctor was discharging me, and I could go home. They helped me to prepare my things, then I got up on my own, put on my gown and shoe covers, and they helped me to do it as quickly as possible, so I could go home. When I left, they said, ‘Good-bye Anto! Don’t come back here! We don’t want to see you here anymore!‘ So, I repeat, that was a really good moment I had, really nice”* (Patient 6)
	**2.4 Connectedness**	“*But I was alone from morning to night and from night to morning… thinking, “Okay. Help, if it’s not good, I’ll go to the intensive care unit. Help, if it’s not good I’ll go to the intensive care unit.”*(Patient 5) “*To see people die like that––that is, in addition to dying, being so isolated, without anyone, without a family member by their side, something that everyone in a moment of illness, of fragility, would like to have.”* (Nurse 4) *“After the patients had only us HPs, the relationship between us and them became closer. For example, they needed us even just to eat. So the concept of dignity has changed during this time. Relatives always remain important, and their absence is felt, but we have tried to do what we can as HPs, to make them feel as good as is possible, so that is another positive aspect. The patient has established a relationship of trust and closeness [with us] in part due to this exclusion of relatives because of COVID.”* (Nurse 7) “*All of the HPs were very attentive from a human standpoint. They were really supportive of the patients, in an extremely empathetic and intense way, and that is a wonderful thing.”* (Physician 3) “*(…) However, many patients that we have seen have been ‘adopted’ (…) patients who felt more and more ‘at home,’ almost.”* (Physician 1)
**Category 3**	**Themes**	**Exemplars and Quotations**
**Dignity in practice**	**3.1 Preservation of Choice**	*“The dignity of the patient (…) as much as I have been able to guarantee it to them (…) begins, first and foremost, with true and transparent information, with giving them the possibility to make choices about their life and not treating them like a child, not hiding anything from them.“* (Physician 3) *“In my opinion, dignity is making the sick person understand why we are treating them, and not just doing things to them without (their) awareness. In my opinion, patients have the right to understand and be aware of their illness, of their own state of health.“* (Physician 2) *“Then you were abandoned to your own devices (…) and you did not know if you were going to stay or if you would have to go into intensive care (…) you were very afraid because you did not know what was happening to you… No one who gives you feedback tells you anything.“* (Patient 5) *“They answered every question I asked very thoroughly, and in terms of answering, they expressed themselves very clearly. I am not a doctor, I do not have great capacity for understanding medical terms, but they explained them to me.“* (Patient 1)
	**3.2 The Body and Small Everyday Acts**	*“I still wasn’t accustomed to this act of being washed. They told me not to worry, that it was their job, like many others. [They were] so very kind, they gave me back my dignity.“* (Patient 3) *“Yes, now and then we shaved him. It was like something from ordinary, everyday life, which was not always possible during the first lockdown, when there was even more confusion (…); now we also know a little bit more about what to do, and we can better transmit a sense of ordinary life to the patients.“* (Physician 2)
	**3.3 Information overload**	“*Yes, I think that (dignity) can also be influenced by others, by the media… Going back to speech in newspapers, it matters a lot, because, when I would enter the rooms, we’d have the TV, and the channels were all talking only about COVID, COVID, COVID, COVID. I’d go in and I couldn’t even make a joke.”* (Nurse 4) “*Before being hospitalized, I said, “I will be cured,” and I didn’t think anymore about the news and that ‘bombardment’ (…) With so much information, you didn’t know what might happen to you.”* (Patient 8) *“So, I was afraid of what the news would tell us every day. It may be true in part, but when you experience it firsthand, you realize that many things are not true.”* (Patient 8) “*Sometimes you entered the rooms (…) (and you would hear), ‘Enough, you are annoying. It’s always the same thing. I do not want to do this thing, because it’s useless anyway,’ and so on. Yes, there were also those episodes, but not many. I don’t know, maybe they were influenced by a transient factor, from the TV. Because, no matter what, everything has an impact (…) so maybe they were conditioned by outside factors and they took it out on us as well.”* (Nurse 4)

## Discussion

This study aimed to examine how dignity is constructed within acute and intensive care settings during the COVID-19 pandemic. This period was characterized by many uncertainties, emergency interventions, and the scarcity of time it allowed for talking with patients. Acute and intensive care settings are often characterized by clinical conditions that do not allow the patient to express themselves. In our project, the dignity-in-care has been translated from an end-of-life PC setting to acute and intensive care. These settings assisted patients with a poor prognosis, but in different ways from the PC setting, considering clinical uncertainties or time-limited.

Our study partly confirmed data from the literature and proposed new ones. Several concepts and themes arose from our study: humanity, reciprocity, connectedness, and relationship, as confirmed by the literature [24].

### “Dignity it and itself”

The category named “Dignity in and of itself” describes concepts related to the definition of dignity as “moral and inalienable good of all human beings regardless of age, social status, ethnicity, religion, gender, physical and mental state” [24]. The reference to the cultural and moral sphere of the person, to be respected, specifies that dignity is inseparable from the person himself, confirming that the “continuity of self” is crucial for the protection of dignity [16–20]. The sense that the essence of who one is continues to remain intact, despite one’s clinical condition, is not a passive way of affirming the person, but a way through which this quality can emerge precisely in the face of a situation that threatens them. HPs and patients suggested being aware that their attitudes and assumptions can influence the way they related to each other [12, 16, 17]. These attitudes are further confirmed by the themes -Dignity as Sense of Intactness and Dignity as Humanity- highlighting the possibility to restore dignity when HPs give the patient his wholeness (looking at him, giving him back autonomy, giving him the information that allows him to choose, allowing him to express his emotions, resigning, etc.): “*More than dignity, it was actual humanity, especially if we consider all that they needed to take care of*” (Patients 6), and “*I believe that if someone lost dignity, to me they would no longer make sense as a person*” (Nurse 4), as confirmed by others studies [15, 17, 18, 20].

### “Dignity in relationships”

The “Dignity in Relationships” category describes the relationship between dignity and how the care relationship is experienced and can be described as “being present with self-awareness”. The care relationship became a space in which HPs recognize the uniqueness of the individual and patients felt like individuals, whose values are to be recognized and respected. These results are consistent with the literature [9–12, 16, 17], confirming that the “continuity of the self” is fundamental to the protection of dignity, even in an emergency setting.

Implementing a person-centered approach requires HPs to ask themselves what has been coined the Patient Dignity Question i.e., what they need to know about patients as individuals to give them the best care possible [25]. Self-reflection and self-care are fundamental if HPs are to avoid “the risk of undignified actions,” as suggested by other studies [26, 27]. The self-care practice involves self-awareness, self-compassion, and implementing various strategies across inner life domains [19, 20].

Our results showed how being isolated was the factor that most undermined patients’ dignity, as reported in other studies [8, 28]. While we originally planned to interview family members, this was not possible, given no patient permitted us to approach their loved ones. None of the patients interviewed gave their consent to family members to participate, preferring not to involve them further. They considered it important to protect them from bringing the painful memory back to the period of their hospitalization. The authors decided to not approached other family members of a different group of patients, because it wasn’t the intent of the current study. Rather, it would explored the same experiences of receiving healthcare during the pandemic from multiple perspectives. Eliciting a separate family cohort would not have accomplished that, and besides, data reflecting on the family experience of COVID-19 is already well reported in the literature [7].

However, the absence of family members might have increased empathy and sharing between patients and HPs. COVID-19 brought the “hospital system” and the “patient system” closer together. In the absence of families, HPs were “forced” to be close to patients, but through small gestures and attention, as a family member would. Similarly, HPs felt that their dignity was recognized by patients when they reported that they were helping and supporting them. In this way, our data suggest that dignity was restored reciprocally: inputs to dignity produce an output of well-being [29].

### “Dignity in practice”

The third category “Dignity in practice” detected that recognizing and affirming the values important to the patient, his beliefs about illness, death, and treatment, and his culture and religion, are all ways to safeguard the dignity of the patient. Both HPs and patients expressed the value of *informing* and *being informed* about clinical conditions, uncertainties, and communication difficulties related to COVID-19. Patients felt their dignity was violated when they were unable to choose therapies e.g., the type of ventilation. This was mirrored by the feelings of HPs who, when they were able to correctly inform patients, perceived that they had respected their dignity.

The small acts of care performed during each brief visit and daily provision of information to patients and family members about the evolution of the disease were “tools” that HPs could use to restore patients’ dignity [16, 17, 19, 20]. HPs and patients felt that information overload could undermine a sense of hope and make a patient feel besieged by negative thoughts, suggesting again the importance of the care relationship that should be constructed at every moment through honest communication.

Several tools have been developed to support HPs in addressing the issue of dignity [13, 15, 30]. However, they require a cognitive and emotional commitment to the patient, something that cannot always be implemented in an acute or intensive care setting. In such circumstances, the use of short open questions such as the Patient Dignity Question can help HPs to understand who a patient is [31].

## Conclusions

Our study partly confirmed data from the literature and proposed new ones, suggesting that dignity should be protected and enhanced by all HPs, not only those in PC or end-of-life but also emergency departments. Interestingly, our data confirm that recognizing and affirming the values important to the patient, his beliefs about illness, death and treatment, and his culture and religion, are all ways to safeguard the dignity of the patient. Moreover, ensuring the best conditions for honest information on the disease and care, and giving emotional and spiritual support are all actions that HPs can act in the care relationship to safeguard the dignity of patients.

### Limitations of the study

Our study was conducted in a single healthcare center, and as such, should be generalizable beyond this study cohort with caution. Moreover, the sample size might appear relatively small, if considered from a quantitative point of view, albeit qualitative studies can typically describe interesting phenomena even when, as in this case, the number of people that lived that experience and described it in the research is relatively small. Lastly, this study was conducted in Italy; hence, further research needs to be conducted in other settings to assess the transferability of the findings across another cultural context.

### Electronic supplementary material

Below is the link to the electronic supplementary material.

Supplementary Material 1

Supplementary Material 2

## Data Availability

The datasets analysed during the current study are available from the corresponding author upon reasonable request.

## Abbreviations

HPs: Healthcare professional
PC: Palliative care
