# Supplementary material for: The meaning of dignity in care during the COVID-19 pandemic: a qualitative study in acute and intensive care
Source: BMC Palliat Care. 2023 Nov 30;22:192. doi: 10.1186/s12904-023-01311-4 (PMC10688038; doi:10.1186/s12904-023-01311-4)
Supplement: Supplementary file 1 — Supplementary Material 1 [file 12904_2023_1311_MOESM1_ESM.docx]

**Supplementary file 2. The Content of the Interviews**

| **Questions for Healthcare Professionals** | **Questions for Patients** | **Questions for Family Members** |
| --- | --- | --- |
| In terms of your own professional experience, how do you define the *dignity of the patient?* | In terms of your own personal experience, how do you define the term *dignity?* In particular, what does *dignity* mean to you in reference to experiences related to COVID-19? | In terms of your own personal experience, how do you define the term *dignity?* In particular, what does *dignity* mean to you in reference to experiences related to COVID-19? |
| Has the meaning of dignity changed as a result of experiences related to COVID-19? | What do you think or feel can enhance dignity? (e.g., words or acts that someone might say or perform, personal ideas or thoughts they might offer, ways of relating, things that you can do, things that you may have read or heard from other people's experiences) | What do you think or feel can enhance dignity? (e.g. words or acts that someone might say or perform, personal ideas or thoughts they might offer, ways of relating, things that you can do, things that you may have read or heard from other people's experiences) |
| Could you tell me what your definition of dignity is based on? (e.g. personal or professional experiences, literature, ideas or personal values, training, reading) | What do you think or feel can undermine dignity? (e.g., words or acts that someone might say or perform, personal ideas or thoughts they might offer, ways of relating, things that you can do, things that you may have read or heard from other people's experiences) | What do you think or feel that can undermine dignity? (e.g., words or acts that someone might say or perform, personal ideas or thoughts they might offer, ways of relating, things that you may have read or heard from other people's experiences) |
| Are there specific experiences you can recall in which a patient’s dignity was supported during the COVID-19 pandemic? | Can you remember any specific times when you felt that your dignity was particularly supported and valued during the experience of COVID-19? | Can you remember any specific times when you felt that the dignity of your loved one was particularly supported and valued during the experience of COVID-19? |
| Are there specific experiences you can recall in which a patient’s dignity was compromised during the COVID-19 pandemic? | Can you remember any specific times when you felt that your dignity was compromised during the experience of COVID-19? | Can you remember any specific times when you felt that the dignity of your loved one was compromised during the experience of COVID-19? |
| Are there specific experiences you can recall in which your dignity as a healthcare professional (physician, nurse) was supported during the COVID-19 pandemic? | Do you believe that dignity is something that can be dependent on others or on a given context? | Are there specific experiences you can recall in which your dignity as a family member was supported during the COVID-19 pandemic? |
| Are there specific experiences you can recall in which your dignity as a healthcare professional (physician, nurse) was compromised during the COVID-19 pandemic? |  | Do you believe that dignity is something that can be dependent on others or on a given context? |
| Do you believe that dignity is something that can be dependent on others or on a given context? |  |  |
