# Supplementary material for: The meaning of dignity in care during the COVID-19 pandemic: a qualitative study in acute and intensive care
Source: BMC Palliat Care. 2023 Nov 30;22:192. doi: 10.1186/s12904-023-01311-4 (PMC10688038; doi:10.1186/s12904-023-01311-4)
Supplement: Supplementary file 2 — Supplementary Material 2 [file 12904_2023_1311_MOESM2_ESM.docx]

**S1 File**

**Consolidated criteria for reporting qualitative studies (COREQ):**

**32-item checklist**

| **No. Item** | **Guide questions/description** | **Reported on Page #** |
| --- | --- | --- |
| **Domain 1: Research team and reﬂexivity** |  |  |
| *Personal Characteristics* |  |  |
| 1. Interviewer/facilitator | Which author/s conducted the interview or focus group? | Two authors (LB and LDP) conducted the interviews. |
| 2. Credentials | What were the researcher’s credentials? E.g. PhD, MD | LB: PsyD  ST: MD, PhD  LDP: PhD  GM: MD  MHC: MD  MCB: MA  MM: MD  MC: MSC |
| 3. Occupation | What was their occupation at the time of the study? | LB: Researcher and psycho-oncologist psychotherapist  ST: Researcher and palliative care physician  LDP: Researcher and Bioethicist  GM: Researcher physician  MHC: Professor and psychiatric  MCB: librarian  MM: physician  MC: nurse |
| 4. Gender | Was the researcher male or female? | LB: Female  ST: Female  LDP: Female  GM: Male  MHC: Male  MCB: female  MM: male  MC: female |
| 5. Experience and training | What experience or training did the researcher have? | The interviewers, moderators, and analysts were experts in qualitative methods. Please, see p.5,6 |
| *Relationship with participants* |  |  |
| 6. Relationship established | Was a relationship established prior to study commencement? | No relationship established prior to study commencement, p.6 |
| 7. Participant knowledge of the interviewer | What did the participants know about the researcher? e.g. personal goals, reasons for doing the research | No knowledge, p.6. |
| 8. Interviewer characteristics | What characteristics were reported about the inter viewer/facilitator? e.g. Bias, assumptions, reasons and interests in the research topic | LB is the PI of the study. |
| **Domain 2: study design** |  |  |
| *Theoretical framework* |  |  |
| 9. Methodological orientation and Theory | What methodological orientation was stated to underpin the study? e.g. grounded theory, discourse analysis, ethnography, phenomenology, content analysis | A qualitative, prospective study by means of semi-structured interviews. Please, see p.4 |
| *Participant selection* |  |  |
| 10. Sampling | How were participants selected? e.g. purposive, convenience, consecutive, snowball | Purposive sampling. Please, see p.4 |
| 11. Method of approach | How were participants approached? e.g. face-to-face, telephone, mail, email | Telephone. Please, see p.5 |
| 12. Sample size | How many participants were in the study? | Five physicians, three nurses, and eight patients. Please, see p.6 |
| 13. Non-participation | How many people refused to participate or dropped out? Reasons? | All the patients and HPS accepted to participate. However, none of the patients interviewed consented for family members to participate. Please, see p.4 |
| *Setting* |  |  |
| 14. Setting of data collection | Where was the data collected? e.g. home, clinic, workplace | Online and by phone- Please, see p.6 |
| 15. Presence of non-participants | Was anyone else present besides the participants and researchers? | No. |
| 16. Description of sample | What are the important characteristics of the sample? e.g. demographic data, date | Gender distribution and age. Please, see Table 1 and 2. |
| *Data collection* |  |  |
| 17. Interview guide | Were questions, prompts, guides provided by the authors? Was it pilot tested? | Yes, they were provided by authors. A multi-disciplinary expert panel designed the interviews, after reviewing the literature on the concept of dignity in terminally ill patients. The interview wasn’t pilot tested. Please, see Supplementary File 2. |
| 18. Repeat interviews | Were repeat interviews carried out? If yes, how many? | No, they weren’t. |
| 19. Audio/visual recording | Did the research use audio or visual recording to collect the data? | The interviews were audio-recorded, p.5 |
| 20. Field notes | Were ﬁeld notes made during and/or after the inter view or focus group? | No, we didn’t made field notes. |
| 21. Duration | What was the duration of the inter views or focus group? | The interviews lasted between 15 and 40 minutes. Please, see p. 6 |
| 22. Data saturation | Was data saturation discussed? | No. |
| 23. Transcripts returned | Were transcripts returned to participants for comment and/or correction? | No. |
| **Domain 3: analysis and ﬁndings** |  |  |
| *Data analysis* |  |  |
| 24. Number of data coders | How many data coders coded the data? | Two authors conducted the thematic analysis based on the verbatim transcripts. The data was validated by two external researchers. Please, see p.5,6 |
| 25. Description of the coding tree | Did authors provide a description of the coding tree? | No |
| 26. Derivation of themes | Were themes identiﬁed in advance or derived from the data? | They have been derived from the data. |
| 27. Software | What software, if applicable, was used to manage the data? | Not applicable |
| 28. Participant checking | Did participants provide feedback on the ﬁndings? | No. |
| *Reporting* |  |  |
| 29. Quotations presented | Were participant quotations presented to illustrate the themes/ﬁndings? Was each quotation identiﬁed? e.g. participant number | Yes, please see Table 3. |
| 30. Data and ﬁndings consistent | Was there consistency between the data presented and the ﬁndings? | Yes (as study authors, we can be biased in the evaluation of this domain) |
| 31. Clarity of major themes | Were major themes clearly presented in the ﬁndings? | Yes (as study authors, we can be biased in the evaluation of this domain) |
| 32. Clarity of minor themes | Is there a description of diverse cases or discussion of minor themes? | No |
